# Wireless Networking-Driven Healthcare Approaches in Combating COVID-19

**DOI:** 10.1155/2021/9195965

**Published:** 2021-12-30

**Authors:** Syed Mohammed BasheeruddinAsdaq, N. Raghavendra Naveen, Lakshmi Narasimha Gunturu, Kalpana Pamayyagari, Ibrahim Abdullah, Nagaraja Sreeharsha, Mohd Imran, Abdulkhaliq J. Alsalman, Maitham A. Al Hawaj, Mohammed Al mohaini, Abdullah A. Alsubaie, Khulod D. Alanzi, Maha S. Alanazi, Amani A. Alanazi

**Affiliations:** ^1^Department of Pharmacy Practice, College of Pharmacy, AlMaarefa University, Dariyah, Riyadh 13713, Saudi Arabia; ^2^Department of Pharmaceutics, Sri Adichunchanagiri College of Pharmacy, Adichunchanagiri University, B.G. Nagar, 571448 Karnataka, India; ^3^Department of Pharmacy Practice, Annamacharya College of Pharmacy, Rajampeta, India; ^4^School of Pharmacy, Management and Science University, Seksyen, 13 Shah Alam, Selangor, Malaysia; ^5^Department of Pharmaceutical Sciences, College of Clinical Pharmacy, King Faisal University, Al-Ahsa 31982, Saudi Arabia; ^6^Department of Pharmaceutics, Vidya Siri College of Pharmacy, Off Sarjapura Road, Bengaluru, 560 035 Karnataka, India; ^7^Department of Pharmaceutical Chemistry, Faculty of Pharmacy, Northern Border University, Rafha 91911, Saudi Arabia; ^8^Department of Clinical Pharmacy, Faculty of Pharmacy, Northern Border University, Rafha 91911, Saudi Arabia; ^9^Department of Pharmacy Practice, College of Clinical Pharmacy, King Faisal University, Al-Ahsa 31982, Saudi Arabia; ^10^Basic Sciences Department, College of Applied Medical Sciences, King Saud Bin Abdulaziz University for Health Sciences, Al-Ahsa 31982, Saudi Arabia; ^11^King Abdullah International Medical Research Center, Al-Ahsa 31982, Saudi Arabia; ^12^Saudi Board of Preventive Medicine, Ministry of Health, Al-Ahsa 31982, Saudi Arabia; ^13^Faculty of Pharmacy, Northern Border University, Rafha 91911, Saudi Arabia

## Abstract

Since its outbreak, the coronavirus (COVID-19) pandemic has caused havoc on people's lives. All activities were paused due to the virus's spread across the continents. Researchers have been working hard to find new medication treatments for the COVID-19 pandemic. The World Health Organization (WHO) recommends that safety and self-measures play a major role in preventing the virus from spreading from one person to another. Wireless technology is playing a critical role in avoiding viral propagation. This technology mainly comprises of portable devices that assist self-isolated patients in adhering to safe precautionary measures. Government officials are currently using wireless technologies to identify infected people at large gatherings. In this research, we gave an overview of wireless technologies that assisted the general public and healthcare professionals in maintaining effective healthcare services during COVID-19. We also discussed the possible challenges faced by them for effective implementation in day-to-day life. In conclusion, wireless technologies are one of the best techniques in today's age to effectively combat the pandemic.

## 1. Introduction

Coronavirus illness (COVID-19) is a respiratory infection that first appeared in Wuhan, China, in 2019. The World Health Organization (WHO) has labeled it a pandemic since its emergence due to widespread transmission across continents [1, 2]. It affects persons of all ages, with the elderly, especially those with comorbid diseases, having a higher mortality rate. People above the age of 80 had a 12 times higher mortality risk than those between the ages of 40 and 59 [3]. On average, women were nearly 0.73 times more likely to be infected with COVID-19 than men. Hence, age and gender are considered socioeconomic inequalities of the COVID-19 pandemic [3]. Overcrowding, race, and ethnicity are some of the other socioeconomic inequalities reported by COVID-19 [3, 4]. Researchers around the world from different fields such as artificial intelligence (AI), biomedicine, pathology, and virology are contributing their work regarding COVID-19 to combat the virus by providing detailed information on virus morphology and its virulence [5]. Technology plays a crucial role in the combat of COVID-19 during the pandemic. AI, the Internet of hospital things (IoHT), deep learning techniques, 5G, and other technologies, such as wireless communication networks, are increasingly being used to combat the pandemic [6].

Wireless technologies such as mobiles, Bluetooth, and Wi-Fi help us communicate with each other without the use of cables. During this pandemic, many countries applied wireless technologies to combat the virus effectively. Countries like the USA, China, and Korea implemented tracing systems integrated with their netizen mobile devices to find their locations during the lockdown [7]. Governments, institutions, and industries depend on social media platforms like Zoom, Skype, and Team Link to communicate with each other in pandemic times. Sensors, drones, and smart helmets are being used in airports, bus stands, and other areas of social gatherings. All these constitute part of wireless technology [7].

Wireless technologies permit access to continuous patient care while maintaining the patient's safety and privacy in a health crisis. This process was achieved through the relaxation of government limitations after the pandemic. Vidal-Alaball et al. explain the role of telehealth services during the COVID-19 pandemic [8]. According to them, wireless technologies can be used as a platform for online consultations, help monitor patients through smart devices, and can aid in avoiding dangerous places with high viral loads using wireless technologies. Bajowala et al. concluded that wireless technologies in the form of telemedicine can be safe and effective for payment of hospital transactions and can be used in the billing and coding areas of hospitals [9]. Blue et al. discussed the role of wireless technologies in the neurology department during the COVID-19 pandemic [10]. They concluded that accurate and effective neuroexamination can be done through the implementation of wireless technologies as telemedicine services. Boehm et al. discussed the importance of wireless technologies around urology wards during COVID-19 [11]. They reported that many patients are willing to take their appointments in hospitals through a wireless platform named “telemedicine.” Bokolo emphasized adopting virtual software platforms for outpatients visiting hospitals during the pandemic timeframe [12]. Their findings concluded that wireless technologies could minimize emergency room visits, safeguard healthcare resources, and decrease the COVID-19 spread by remotely treating patients during and after the COVID-19 pandemic. Zhou et al. constructed a 5G network integrated with wireless technologies in a new model in the hospital cabin [13]. This model solves the different problems faced by hospitals, such as Internet access, data filling, and file sharing and storage. The special architecture within this model helps in updating patient records, nurses' activities, and radiologists' scans and reports. Janjua et al. described the use of wireless technologies during COVID-19 in hospital areas [14]. According to them, wireless technologies improve the capacity of data usage by using high frequency bands and improve hospital coverage using various ad hoc networks and device-to-device connections. Saeed et al. in their study discussed the possible advantages of wireless communications during the COVID-19 times to improve the country's economy [15]. They can boost online activity for a smooth flow of e-commerce, protect high-risk individuals from virus spread with touch-less solutions, and reduce viral growth by flattening the curve during lockdown times. Al-Humairi and Kamal discussed the prospective use of wireless technologies towards the building of monitoring systems [16]. They reviewed the possible uses of thermal scanning technologies in buildings, the use of Swann security cameras, and the integration of infrared thermometer devices developed by wireless technologies to monitor the public vitals and body temperature in buildings and public places. Cervino and Oteri discussed the importance and usage of telephone triage for COVID-19 patients in medical settings [17]. They emphasized that telephone triage has the capability to identify COVID-19 patients with disease symptoms, help examine the patient's general health condition, and identify the risks associated with COVID-19 patients. Hence, telephone triages are considered operative filters to prevent the spread of COVID-19 infections. Therefore, wireless technologies are one of the common approaches implemented in the contemporary COVID-19 pandemic to control the virus' spread and improve public safety.

In this review article, we mainly focus on applications of various wireless technologies for the public and patients during the COVID-19 pandemic that can further help physicians, the public, and other healthcare professionals gain awareness and ideas regarding the importance of informative technologies to prevent the spread of pandemics.  Section 3 deals with wireless technologies' role in the pandemic for the public, healthcare professionals, and remote applications. In Section 4, we discussed the possible challenges faced by wireless technologies, along with solutions for effective implementation. Finally, in Sections 5–7, we discussed the limitations of this study and provided the overall conclusion on wireless technologies and their impact during the COVID-19 crisis.

## 2. Methodology

The information was gathered from published literature by searching scientific databases for specific topics and key terms (Table 1). To find relevant scientific data, researchers used advanced PubMed searching with MeSH keywords.

Wireless communications, COVID-19, pandemics, applications, and challenges were used in a search strategy for publications published between 2019 and 2021. The screening of titles and abstracts was done manually. These articles' full texts were then reviewed for possible inclusion and exclusion criteria. Case studies and unauthored proofs were excluded.

## 3. Wireless Technology Applications

In the current pandemic situation, the main challenge for healthcare organizations is to prevent the virus' spread and help the public maintain safe health by following adequate preventive and control measures. In this section, we discussed the applications of wireless technologies to prevent COVID-19 spread. As per issued guidelines by WHO, measures such as avoiding group gatherings, maintaining social distance, and tracing of netizens are important measures to prevent the rapid viral spread. Here we discussed wireless technologies to prevent the viral spread by tracing indoor and outdoor activities of the public.

### 3.1. Outdoor Tracking

A different variety of wireless technologies, such as drones, mobile phones, and global positioning systems, is used to monitor the viral spread in outdoor areas [18]. In cities with a denser population, network drones are used to monitor the crowds for social distance maintenance. These drones also help to raise awareness among the public regarding social distance measures [6]. The pandemic drones are also used to monitor the variations in body temperature, changes in normal physiological body functions, and the presence of flu, coughs, and sneezes in public places [19, 20]. Once the information is collected from such suspicious people, it is transferred to higher authorities for appropriate actions. Drones with 5G connectivity can facilitate this process even faster because of faster Internet connectivity and low latency. Satellite communications are another huge advantage in monitoring and remodeling the COVID-19 spread. COVID-19 may spread rapidly in areas with huge populations; hence, this can be monitored using geospatial data and satellite images to identify the populations at risk of getting COVID-19 [21].

Another aspect of fighting the COVID-19 pandemic is identifying individuals with COVID-19 infection. To make the process easier, social platforms such as Google and Facebook have initiated a venture on GPS-based user data. This will help to track the infected people with their current location [22, 23]. A contract tracing system developed by Google and Apple that uses Bluetooth signals to identify nearby smartphone users and sends alarming signals if they come within a certain distance of COVID-19 patients. This also reduces the transmission of the virus [24]. When the infected person comes out, there is a huge chance of viral spread from the infected patient. So, to prevent this and effectively monitor the patients, wearable bands are used. These are cost-effective and provide accurate results. They are connected to a patient smartphone application with Bluetooth and sensors that can help track the identity of a person [25].

### 3.2. Indoor Monitoring

In the outdoor environment, technologies such as GPS and Bluetooth are being used to maintain social distance and prevent the spread of the virus. However, it is challenging to maintain the social distancing guidelines within the house and indoors due to the unavailability of such technologies. Hence, novel technologies are needed to prevent indoor viral spread to overcome the problem. Those include Wi-Fi, visible light, Bluetooth, and radio frequency identification. These have proved to be promising solutions for self-isolated people [26]. For example, a new technology called proximity tracing is being used during the COVID-19 pandemic times to identify an individual's presence. This helps the public maintain the work environment distance monitored indoors [27]. This proximity test has a tag that should be worn by the workers. It can work in both an active and passive manner. The active manner can alert the workers within the working environment when they come close to each other by violating the social distancing guidelines. The passive approach can provide information to tracing authorities when the staff is infected with the COVID-19 virus [27].

An app called “Social Monitoring” was introduced by the Russian government and made mandatory for its netizens to install it on mobile devices. Once the app is installed, patients are asked to scan for the quick response code whenever they leave the quarantine place [28]. Another wireless technology is the Easy Band, a wearable device. It provides an alarming sound when there is a violation of social distancing norms between two people [29]. Apart from these indoor applications, they prepare the network graphs using sensing measurements from the proximity users to know the contract tracing and working distance between the people [30].

### 3.3. Healthcare Applications

**Figure 1:**
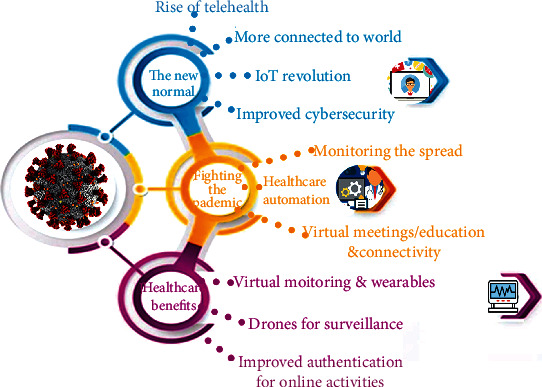
Applications of wireless technologies for COVID-19 in various healthcare domains.

The outbreak of COVID-19 increased the trend of utilizing wireless communications and 5G networks in healthcare (Figure 1). Many hospitals extensively use Wi-Fi networking services to permit a better connection and allow better response times for the public and local communication. Medical robots are used to deliver drugs and check the patient's vitals such as body temperature and blood pressure to disinfect the rooms of hospitals to prevent the spread of viral infections [31]. These robots, connected with wireless technology integrated with 5G services, can collect patient data and share it with remote data centers to improve the efficacy of healthcare systems [32]. The information exchange from these robots needs to be accurate and low-latency communication as provided by these wireless technologies. To improve patient care, China has developed an advanced hospital system that is integrated with wireless and 5G connectivity [32].  Table 2 summarizes the wireless technologies that were utilized to deliver better healthcare services to patients during the COVID-19 pandemic crisis.

Fiorillo et al. emphasized the need for a protocol for prevention of COVID-19 spread in medical settings, especially in dental offices. Dental wards are commonly used as a potential source for various microorganisms due to the increased likelihood of the formation of microbial films [33]. Therefore, dental units and medical instruments used in these areas are to be sterilized with 0.1% sodium hypochlorite or 0.5% hydrogen peroxide. In addition to these, the establishment of air purifiers and aspirators in dental clinics can reduce the load of microorganisms present in the air [33]. D'Amico et al. further discussed the need for management of COVID-19 patients in dental wards. Their work emphasized the importance of the usage of telephone triages, the maintenance of social distance among the patients in waiting rooms, and the role of protective personal equipment (PPE) in the prevention of COVID-19 spread in dental wards [34].

Lu et al. described the use of new wireless technologies to manage the pain of patients during the COVID-19 crisis. As most of the nonemergency procedures are halted during this time, it becomes difficult for the patients who are implanted with spinal cord devices to tolerate the pain [35]. They developed a remote programming system that helps healthcare professionals monitor such patients through video programming and deliver safe palliative medicine to such patients implanted with spinal cord devices [35]. Silva et al. discussed the importance of wearable patches to monitor the vitals of COVID-19 patients. They are used in the long term because they can improve patient safety and treatment outcomes [36]. This type of wearable patch also reduces the pressure on healthcare professionals as the patients are monitored remotely. Besides this, they also help to gather a huge number of patients' data in a timely manner so that treatment outcome is enhanced [36]. Ni et al. discussed the association of body vital signs with cloud data infrastructure to monitor the COVID-19 patients. This infrastructure program primarily examines measurements such as coughs and vocal cord changes that occur as the disease progresses [37]. They can also link the association with the frequency of coughing and droplet production to COVID-19 disease severity. Hence, continuous monitoring of such vital parameters in COVID-19 patients can improve the therapeutic outcomes [37]. Zhang et al. developed a wireless stethoscope that can monitor the pulmonary vitals in COVID-19 pneumonia conditions [38]. This device can record various lung sounds. This study concluded that Velcro crackle lung sounds are predominant in COVID-19 pneumonia cases and poor diagnosis was recorded in severely ill patients while the presence of auscultatory sounds in moderate and mild disease patients improved the disease prognosis [38]. Dini et al. developed a wireless device called Lung Ultrasound that can monitor the lung injury in bedridden patients. This study included 150 subjects with COVID-19 pneumonia [39]. The lung ultrasound wireless device had a 79% sensitivity of detecting the COVID-19 positive cases through a nasopharyngeal swab. This device identified that 73% of patients included in the study were positive for the COVID-19 virus (*p* = 0.016). Hence, Dini et al. concluded that use of lung ultrasound integrated with wireless portable sensors could be a better option to estimate the disease condition and lung injury among patients with COVID-19 pneumonia [39].

In their study, Kancharla et al. described the importance of heart vital monitoring during the COVID-19 pandemic in patients with existing arrhythmias and QT intervals [40]. It included 82 inpatients with the use of mobile patch-based technology. Findings by Kancharla and Estes concluded that there was a reduction of 595 minutes of viral exposure in hospital staff with the implementation of wireless technologies to monitor cardiac vitals. It also increases the staff presence in emergency departments. Hence, patch-based mobile technology reduces infection as there is no direct patient contact with the physician and can be beneficial to COVID-19 patients with existing heart abnormalities [40]. Yilmaz et al. developed a sound acquisition module that integrates within the patient's garments and helps minimize stethoscope use. This technology provides an option for respiratory ill patients to benefit from long-term vitals monitoring during the COVID-19 crisis [41].

### 3.4. Remote Healthcare

Due to restrictions imposed on travel, remote health settings are globally common in areas with a lack of healthcare facilities. Hence, healthcare for the people in those areas can be provided in two ways. One is through telemedicine, and the other is through remote health monitoring. In platforms like telemedicine services, doctors make use of smartphone teleconferences or scrutinize the electronic health records of patients for appropriate diagnosis and evaluation of treatment outcomes [14]. Such a type of healthcare facility is available in houses or basic healthcare centers in the presence of paramedical staff. In the present world, many physicians are using teleconferences to connect virtually with their patients without physical contact. However, as digital technologies are evolving continuously, other options such as holograms and holographic presentations will be in use soon [14]. Telemedicine and Internet of medical things technologies such as wearable devices and wireless body networks have begun to be used exclusively to provide healthcare facilities to people in rural areas. IoHT is used as an active tracker of a patient's condition. Biomedical sensors that provide physiological activity and record vital parameters are used in IoHT-based healthcare devices that collect data, analyze the information recorded, and regularly monitor the patient's condition [14, 36]. They can minimize stress levels and record the amount of physical activity done by the person each day. The best examples of this are blood pressure monitoring devices, pulse recording devices, pacemakers, hearing aids, and smartwatches. One such application is the Biostrap, a wearable device that monitors heartbeats. They can help the patient administer the appropriate drug or medication for an existing disease and act as an alarm tool [14], so that, without the need for a caregiver, the patient can take his medication. For all these reasons, IoHT is also known as “Smart Health” or “Smart Technology.” In a similar manner, the implementation of real-time health tracking systems helps to improve the elderly patient's health by tracing out emergencies through the sensors' integration, thus providing the patient medical care. Despite these benefits, the implementation of technology is a major limitation, especially for the elderly, as they are not aware of the information technologies. In addition, to regular dose adjustments for chronic disease patients such as diabetics and hypertensive patients, the implementation of teleconferencing or virtual modes, is the easiest way to provide health facilities for regular care. Despite these many advantages, informative technologies and wireless communications need high security with privacy guidelines to store patient data [6].

Since the increase in coronavirus cases, rural healthcare has been given prime importance to people to reduce the infection risk associated with the virus. Rural healthcare is completely dependent upon the network's facilities and infrastructure to provide better health outcomes. Due to this, many information technologies such as massive connections, ultrapower connections, and low-latency tactile Internet-dependent remote facilities have not yet been approved [31].

### 3.5. Technologies Used in Digital Tracing

One of the most important precautionary guidelines to prevent the COVID-19 spread is the maintenance of social distance. Many informative technologies are used to achieve the goal of effective social distance maintenance. Such technologies are Wireless Fidelity (Wifi), Quick Response (QR) codes, the Global Positioning System (GPS), and ZigBee.

#### 3.5.1. Wifi

This informative technology is extremely useful for tracing coronavirus-infected patients, particularly during the self-isolation period. These are used to monitor patients in buildings, hospitals, and other congested areas. They provide high, extreme accuracy of the inner environment in contrast to other existing devices. In this informative technology, there is the presence of the wireless connections associated with the sensors, by which signals are shared with the government and healthcare authorities to maintain updated information records on coronavirus cases [42]. These are very useful in public places, especially in railways and airports. It is also cheaper to maintain the services and maintain them.

#### 3.5.2. Bluetooth

Another wireless technology used in the control of the coronavirus pandemic is Bluetooth. It is present in almost every smartphone. There are several patterns of Bluetooth devices. Among them, Bluetooth low-energy protocol devices are more popular because of their low-energy expenditure and less energy is used. As a contract tracing option, these are switched on every time to trace the information. One of the big advantages of the use of Bluetooth devices is that they can be connected to many devices without the need for an access point. Government authorities in Singapore manufactured an app called Blue Trace. The protocol implemented in this app is so simple and clear that when this app comes in close connection with another app, it can save the data manually in its database [43]. Later, this information is shared with the government authorities to maintain the records [23].

#### 3.5.3. GPS

The GPS uses satellite systems to trace the individual's identity. The option of GPS is provided in all smartphones, where it must be enabled to track the patient information. In the conditions of the pandemic, such options are enabled on public smartphones for contract tracing. Another advantage of the implementation of GPS tracking is that it can minimize direct physical contact between the person and another person. For example, when customers purchase products online, those products are delivered to the respective houses of the public through unmanned aerial vehicles. Many big stores incorporate this GPS technology to deliver products. Therefore, social distance among the public is effectively maintained through the GPS tracers. Many effective solutions are deployed by GPS to geolocate the public in self-isolation. The use of smartphones enables GPS trackers. It can record information on individuals' movements and locations and then share the respective data with government officials [23].

#### 3.5.4. QR Codes

Another method to trace the individuals is by QR code scanning. Here, a person can take a picture virtually at multiple locations. This picture is scanned and analyzed by the mobile databases and provides an option for geolocation. For example, if the public is tested positive for the coronavirus, then the information of the respective person can be traced easily as it provides geological location. Apart from this, places visited by infected people can also be traced. Therefore, informative technologies like QR codes are being used worldwide to restrict the coronavirus pandemic.

#### 3.5.5. ZigBee

This is yet another effective technology that is primarily used in maintaining social distance during pandemics. It is a low-cost and low-energy network information technology. These devices are able to communicate with other devices within a 20-meter range. This device consists of a hub internally, which can be used to identify the user's location. As a result, this technology can be used to maintain effective social distance guidelines in crowd-pulling areas [44].

Recent technologies such as 5G also play a crucial role in the control of the COVID-19 pandemic. This technology is typically used for the construction of cabin hospitals during pandemic times. We usually need a cabin or hospital area with a clinic data network. A lease line is provided by an Internet service provider to connect with the hospital network. After this set up is completed, patients who are visiting the clinic will find their applications are connected to this network and all their records are saved to maintain the electronic records [13].

Blue et al. discussed the importance of wireless technologies, especially telehealth services, during the pandemic. According to them, these information technologies are useful for providing general patient examinations such as vital sign evaluations and physical examinations through webcams without direct physical contact [10]. Boehm et al. used wireless technology in the urology departments of medicine. They concluded that nearly half of the patients visiting the clinic were interested in opting for the choice of telemedicine during the pandemic. They also reported a decline in viral spread through the implementation of wireless technology resources in hospital settings [11]. Bokolo discussed the utilization of wireless approaches in providing outpatient care in hospitals during and after the COVID-19 pandemic. They concluded that informative technologies could decrease the diagnosis time and improve patient care, hence they can act as a proactive measure, especially in pandemic times [12]. In their study, Contreras et al. reported the importance of informative technologies and the change brought by them with the pandemic. They concluded that wireless approaches such as telehealth services and 5G data connectivity will play a key role soon of better patient health care [45]. Chamola et al. concluded that wireless technology services are used in the identification of coronavirus cases, community spread, and diagnosis, especially in molecular tests with the use of sensors for rapid results. They also concluded that wearable devices are used to monitor the body vitals, respiration rate, and saturation rates that are considered important parameters in coronavirus infected patients [6]. With the advent of wireless technologies and the development of sophisticated hospitals, resources have led to the use of telerobots in hospital settings in pandemic times. A human installs a user interface option on this robotic device to control it remotely. These are in use, especially to disinfect patients and the public in hospital settings with the sanitizers. They are also used to undergoing minimal surgeries, especially where a physician cannot operate on the patient due to infection risks in pandemic times [31]. The best example of this is the da Vinci robotic system. Another advantage of the use of robotic services as a part of wireless technologies is that during pandemic times, healthcare professionals usually need personal equipment such as personal protective equipment to reduce the risk of infection and being affected by viruses. Hence, the implementation of such robotic devices has decreased the need for protective equipment, and it can be further dispensed to the public whenever needed. Apart from these healthcare needs, wireless technologies help the public leverage both virtual and augmented realities in pandemic times. As these can be utilized for virtual interfaces, it can help to reduce the social isolation feelings in the minds of patients [31].

### 3.6. Special Advantages of Wireless Technologies

**Figure 2:**
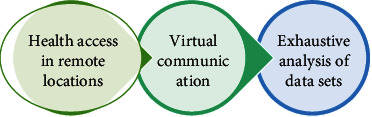
Key benefits of wireless technologies.

The special advantages of wireless technology are presented in Figure 2.

1. With the implementation of virtual meetings, all the patients are examined at home. Therefore, that physician can get a clear idea about the mental status of that person as it might vary in hospital settings
2. Other social histories can be carefully evaluated by the physician, such as the living environment and family interaction
3. This can also yield information about social habits such as alcohol use and tobacco use
4. Reduction in travel charges for the patients who daily visit the hospitals

## 4. Wireless Technology-Related Challenges

In the fight against the COVID-19 pandemic, we cannot ignore the beneficial role of wireless technologies in public safety and healthcare. However, apart from the positive outcomes, there are also challenges associated with wireless technologies, such as privacy, security, and misinformation. Therefore, this section deals with possible challenges and their solutions.

### 4.1. Privacy

Despite the use of contract tracing technologies to prevent viral spread, they also invade public privacy. The user's location is easily accessed by these applications and is used for government record purposes. Human rights activists warn that the use of these applications could manipulate the surveillance guidelines in the coming future. Hence, a few questions have to be addressed by the government authorities before the implementation of such applications. They are as follows:

1. Are users aware of the information they have collected, and can they delete it once the pandemic has ended?
2. How long and who can access this information?
3. Guidelines for sharing the information

Apart from this, drones used for aerial surveillance purposes monitor the social distance between the public in mass gatherings. This raises a general question and breaches the security concerns of the public. Because it can be an infringement on individual liberties [46].

Mobile phone data collected from the public, such as personal location, can prevent the spread of the virus. However, it also poses a risk to individual privacy as the data was collected by government authorities for surveillance. So, to overcome this problem, Bluetooth low-energy technology was used in some countries [47]. With this technology, when two people come close to each other, contract-tracing apps record their identities. They also record the individual location and time of proximity between the users. This information is stored in the device or shared with government authorities as a part of the COVID-19 surveillance program. In the future, when the patient is infected with COVID-19, this information will be shared among all the users as a precautionary measure to protect them from infection. If the user identity is not recognizable and the only information shared is that the process is viable; if not, it leads to a deviation of public privacy [47].

### 4.2. Security

The unequalled utilization of mobile phones during the pandemic outbreak increases the cybersecurity risks for the public. According to Akamai's most recent report in March 2020, overall Internet traffic has increased by 30%. This increased use of the Internet during the pandemic lockdown globally increased the cyber security risks, malicious emails, phishing information related to COVID-19 and the circulation of fake information during the pandemic [48]. Apart from this, many business institutions have started working remotely, which makes the authentication process a challenging factor. All the organization's accounts are transferred online during the pandemic to promote their goods and services among the public. This also increased the cyber-attack. All the applications in the present day are completely automated. This can also lead to an increase in cyber security attacks. Novel digital technologies aroused during the pandemic are at an increased risk of cybersecurity attacks. Hence, necessary actions must be taken to prevent cyber security attacks.

Since the start of COVID-19 pandemic, work from home has become a common phenomenon in all organizations to run services without any failure. This working culture has become a boon for cyber security criminals. Therefore, to prevent malicious cybersecurity attacks, trusted cybersecurity infrastructure considerations are being put into use. They provide awareness among their users on the following aspects [49]:

1. Raising awareness among its users related to COVID-19 fraud
2. Updating and checking the Internet services of organizations regularly
3. Making communication transparent with the company staff
4. Developing safe and secure tools for data transfer

Hence, these are some of the measures taken to prevent cybersecurity attacks during pandemics. Cybersecurity staff and public services can join hands to reduce this fraudulent risk. Apart from those, other limitations such as wider reach, lack of smartphone applications, improper user applications, scalability problems, and transparency of wireless technologies still exist.

## 5. Limitations

We analyzed the published data from the last two years and missed some information that was available earlier. Secondly, only PubMed and Google Scholar were used as sources of information. Third, the lack of statistical analysis prevents us from determining the study's significance. Finally, the data reported in this publication came solely from healthcare areas of interest that use wireless technologies.

## 6. Conclusion

The coronavirus pandemic's rapidity, risk, and severity ushered in a slew of new developments. This event demonstrates the value of healthcare workers and personnel. This eruption sparked debate about the employment of innovative informing approaches and their paradigms in healthcare settings, with the goal of improving patient care while also reducing viral spread. As a result, all international and national organizations, as well as countries, have embraced information technology.

Wireless technologies are critical in the fight against the COVID-19 outbreak and in restoring normalcy to the situation. They can be used in a variety of situations, including surveillance, hospital care, business administration, pharmaceutical chain management, dental clinics, and so on. When similar technologies were used in the early phases of a pandemic, better results were seen in terms of virus propagation. However, there are issues with this application, such as privacy and security concerns, that must be handled depending on how this technology is used in a specific industry.

## 7. Prospects

This work paves the way for future researchers to see the flaws in the wireless technologies discussed above to improve their applications. Apart from this, there is a chance to deploy wireless technologies and telemedicine services in hospitals to provide continuous health facilities for the public even in pandemic times and to minimize the risk of being infected with viruses.

## Figures and Tables

**Table 1:** PICO (Patient, Intervention, Comparator, and Outcome) for designing our question.

Parameter	Included	Excluded
Patient	COVID-19	Other diseases
Intervention	Wireless technologies	Big data
Comparator	Public and healthcare sectors	Industrial, economic sectors, and others
Outcome	Applications and challenges with solutions	—

**Table 2:** Summary of studies related to wireless technology applications in healthcare during the COVID-19 crisis.

Study	Technology type	Application
Lu et al. [35]	Wireless programming system	To deliver safe and effective programming operations for implantable spinal cord stimulation device patients remotely.
Silva and Tavakoli [36]	Wearable biomonitoring patches	Helps to continuous monitoring of patients remotely and ultimately reducing the burden on hospitals.
Ni et al. [37]	Wireless mechanoacoustics	Record coughing frequency and intensity in COVID-19 patients during the disease course.
Zhang et al. [38]	Wireless stethoscope	Auscultator's characters in COVID-19 patients are analyzed in hospitals and indoor settings using this wireless stethoscope.
Dini et al. [39]	Wireless lung ultrasound	It diagnoses lung injury in COVID-19 patients and helps nursing residents to monitor the patients.
Kancharla and Estes [40]	The mobile cardiac monitoring device	It detected the abnormal fluctuations in echocardiography of COVID-19 patients.
Yilmaz et al. [41]	The wireless wearable acoustic transducer	To monitor long-term health benefits of respiratory ill patients with COVID-19.

## Data Availability

The data used to support the findings of this study are included within the article.
